# The Role of TNF-α in Mice with Type 1- and 2- Diabetes

**DOI:** 10.1371/journal.pone.0033254

**Published:** 2012-05-11

**Authors:** Maria Koulmanda, Manoj Bhasin, Zuheir Awdeh, Andi Qipo, Zhigang Fan, Dusan Hanidziar, Prabhakar Putheti, Hang Shi, Eva Csizuadia, Towia A. Libermann, Terry B. Strom

**Affiliations:** 1 Departments of Surgery and Medicine, Transplant Institute, Beth Israel Deaconess Medical Center, Harvard Medical School, Boston, Massachusetts, United States of America; 2 BIDMC Genomics and Proteomics Center, Division of Interdisciplinary Medicine and Biotechnology, Beth Israel Deaconess Medical Center, Harvard Medical School, Boston, Massachusetts, United States of America; 3 Pulsar Clinical Technologies Inc., Cambridge, Massachusetts, United States of America; 4 Department of Internal Medicine, Wake Forest Health Sciences, Winston-Salem, North Carolina, United States of America; La Jolla Institute for Allergy and Immunology, United States of America

## Abstract

**Background:**

Previously, we have demonstrated that short-term treatment of new onset diabetic Non-obese diabetic (NOD) mice, mice that are afflicted with both type 1 (T1D) and type 2 (T2D) diabetes with either Power Mix (PM) regimen or alpha1 antitrypsin (AAT) permanently restores euglycemia, immune tolerance to self-islets and normal insulin signaling.

**Methodology and Principal Findings:**

To search for relevant therapeutic targets, we have applied genome wide transcriptional profiling and systems biology oriented bioinformatics analysis to examine the impact of the PM and AAT regimens upon pancreatic lymph node (PLN) and fat, a crucial tissue for insulin dependent glucose disposal, in new onset diabetic non-obese diabetic (NOD) mice. Systems biology analysis identified tumor necrosis factor alpha (TNF-α) as the top focus gene hub, as determined by the highest degree of connectivity, in both tissues. In PLNs and fat, TNF-α interacted with 53% and 32% of genes, respectively, associated with reversal of diabetes by previous treatments and was thereby selected as a therapeutic target. Short-term anti-TNF-α treatment ablated a T cell-rich islet-invasive and beta cell-destructive process, thereby enhancing beta cell viability. Indeed anti-TNF-α treatment induces immune tolerance selective to syngeneic beta cells. In addition to these curative effects on T1D anti-TNF-α treatment restored in vivo insulin signaling resulting in restoration of insulin sensitivity.

**Conclusions:**

In short, our molecular analysis suggested that PM and AAT both may act in part by quenching a detrimental TNF-α dependent effect in both fat and PLNs. Indeed, short-term anti-TNF-α mAb treatment restored enduring euglycemia, self-tolerance, and normal insulin signaling.

## Introduction

A similar T cell dependent autoimmune process directed against insulin producing beta cells creates type 1 diabetes (T1D) in man and the clinically relevant non-obese diabetic (NOD) mouse model [1], [2]. Moreover, new onset T1D occurring in NOD mice is associated with a type 2 diabetes mellitus (T2D) like state, characterized by defective insulin signaling and thus insulin resistance [3], [4], [5]. In the NOD model, robust expression of pro-inflammatory cytokines within tissues in which insulin directs disposal of glucose appears responsible for the defects in insulin signaling and insulin triggered disposal of blood glucose [4], [5].

While many treatments prevent the development of diabetes in NOD mice [2], few therapies have succeeded in restoring long-term drug free euglycemia and immune tolerance to beta cells in overtly diabetic NOD mice [5], [6], [7], [8], [9]. The beneficial effect of anti-CD3 mAb in NOD mice served as the basis for initiating clinical trials in which anti-CD3 treatment produced remissions in select human subjects with new onset T1D [10], [11]. In our laboratory, treatment with either the Power Mix (PM) regimen consisting of IL2.Ig, mutant antagonist-type IL15.Ig, and rapamycin [5] or alpha1 anti-trypsin (AAT) [4] permanently restores euglycemia, self-tolerance to islets and also eliminates insulin resistance and defective insulin signaling in the NOD model [4], [5]. To search for relevant therapeutic targets in new onset diabetic NOD mice, we have applied genome wide transcriptional profiling and systems biology techniques to examine the impact of PM [5] and AAT [4] regimens upon pancreatic lymph nodes (PLN), a disease relevant immune site, and fat, a site for insulin-dependent glucose disposal.

As noted herein tumor necrosis factor alpha (TNF-α) immerged as a potential therapeutic target for new onset diabetes. Paradoxically, long-term treatment with tumor necrosis factor alpha (TNF-α) [12] as well as short-term treatment with anti-TNF-α [13] prevent the later development of diabetes in NOD mice. Transgenic mice that express TNF-α solely in their islets develop T1D more rapidly than wild type NOD mice [14]. Some advocate therapy with TNF-α or TNF-α inducers [15], [16] as treatment for overt T1D. Does neutralization of TNF-α confer benefit or intensify T1D related autoimmunity in the therapeutically challenging and apparently clinically predictive model of new onset overt diabetes in NOD mice?

## Results

### Microarray and network based analysis of PLNs and fat isolated from AAT and PM treated NOD mice identifies TNF-α as a candidate target focus hub for reversing diabetes

**Figure 1:**
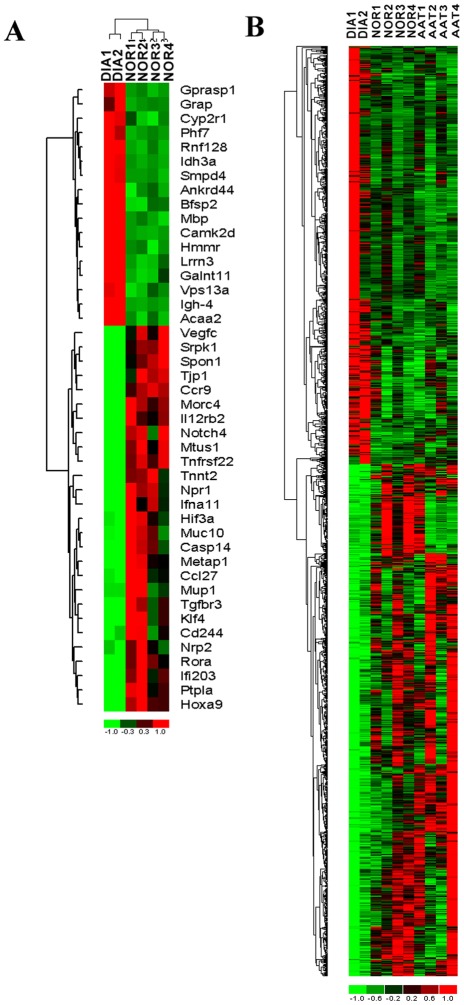
Counter-regulation of gene expression by AAT or PM as determined by DNA microarrays of normal (NOR), diabetic (DIA) and AAT or PM treated PLNs and fat tissues from NOD mice. The genes were identified in a supervised analysis using an absolute fold change of 2 and P value<0.05. **A**) Hierarchical cluster analysis of top transcripts that are differentially expressed in PLNs extracted from diabetic mice vs. normal non-diabetic control mice. **B**) Cluster analysis illustrating the transcripts that are significantly counter-regulated by AAT treatment in PLNs. The columns represent the samples and rows represent the genes. Gene expression is shown with pseudocolor scale (−1 to 1) with red denoting high gene expression levels and green denoting low gene expression levels of genes.

We hypothesized that gene expression changes occurring upon onset of diabetes in NOD mice and reversed by different short-term treatments that cure T1D/T2D may identify relevant therapeutic targets. We performed transcriptional profiling in combination with systems biology analysis on fat and PLNs obtained from normoglycemic NOD (i.e., NOR), new onset T1D/T2D NOD (DIA), as well as AAT and PM (only fat) treated mice. We first identified the transcriptional changes occurring upon new onset of T1D by comparing new onset T1D/T2D fat, a site for insulin directed glucose entry, and PLN, a relevant immune system site, gene expression profiles to fat and PLN from normoglycemic mice. After preprocessing of gene expression data, a total of 1,813 and 4,262 transcripts were identified as significantly differentially expressed (fold change or FC>2 and P value<0.05) in fat and PLNs, respectively, in diabetic as compared to control normal mice. These gene signatures are designated as the “Fat T1D/T2D signature” and the “PLN T1D/T2D signature”. The top differentially expressed genes in PLNs are depicted in **Fig. 1A**, and hierarchical clustering of these genes shows clear distinction between normoglycemic and diabetic mice.

To identify the transcriptional changes induced by AAT or PM treatment that reverse the diabetic phenotype, we performed an analysis to identify the transcripts within the “Fat T1D/T2D signature” and the “PLN T1D/T2D signature” that are counter-regulated by the two treatments using K-means clustering of differentially expressed transcripts. K-means clustering patterns that depict the different degrees of counter-regulation induced by the treatments on transcripts differentially expressed in diabetic (DIA) vs. normal (NOR) NOD mice were identified. In fat tissue, 238 transcripts were counter-regulated by both AAT and PM treatment by a magnitude of FC>2 and P value<0.05 (**Table 1**). In PLNs, 1,367 transcripts were counter-regulated by FC>2 and P value<0.05 in the AAT treatment group as compared to diabetic mice (**Fig. 1B**). These results clearly demonstrate that a significant portion of genes dysregulated during diabetes onset are reversed by AAT or PM treatment in both fat and PLNs suggesting that these sets of counter-regulated genes are critical for diabetes development and have to be reversed in order to restore normoglycemia.

**Table 1:** List of fold changes of the top 30 genes out of 238 genes counter-regulated by AAT and Power Mix treatment in fat.

SYMBOL	GENE NAME	Treatments		
		Diabetes	AAT	Power Mix
Ppbp	pro-platelet basic protein	−13.4	4.1	6.5
Net1	neuroepithelial cell transforming gene 1	−11.1	7.7	14.7
Pck1	phosphoenolpyruvate carboxykinase 1, cytosolic	−9.3	12.6	13.6
Cd24a	CD24a antigen	−5.8	5.4	3.5
Egr1	early growth response 1	−5.4	6.8	9.5
Rsad2	radical S-adenosyl methionine domain containing 2	−4.3	3.8	2.5
Txnip	thioredoxin interacting protein	−3.9	3.6	4.0
Ptp4a1	protein tyrosine phosphatase 4a1	−3.6	2.7	4.3
Crls1	cardiolipin synthase 1	−3.6	2.2	4.1
Mycl1	v-myc myelocytomatosis viral oncogene homolog 1	−3.0	2.3	2.6
Bnip3l	BCL2/adenovirus E1B interacting protein 3-like	−3.0	2.2	2.0
Ube2d3	ubiquitin-conjugating enzyme E2D 3	−2.9	2.2	3.2
Isg20	interferon-stimulated protein	−2.7	2.3	2.2
Ikbkg	inhibitor of kappaB kinase gamma	−2.6	2.6	2.9
Ccng2	cyclin G2	−2.5	2.5	2.4
Psmd7	proteasome 26S subunit	−2.5	2.0	2.0
Pdk4	pyruvate dehydrogenase kinase, isoenzyme 4	−2.3	4.4	4.2
Bcap31	B-cell receptor-associated protein 31	2.3	−2.4	−2.5
Ccnd2	cyclin D2	2.5	−2.7	−2.4
Chst1	carbohydrate (keratan sulfate Gal-6) sulfotransferase 1	2.6	−2.1	−2.3
Acaca	acetyl-Coenzyme A carboxylase alpha	3.0	−4.5	−3.0
Ccnl2	cyclin L2	3.1	−2.2	−3.1
Mrc2	mannose receptor, C type 2	3.1	−2.1	−3.1
Igfbp5	insulin-like growth factor binding protein 5	3.7	−2.6	−3.7
Rpn1	ribophorin I	3.7	−2.1	−3.6
Rhof	ras homolog gene family, member f	3.9	−3.2	−3.9
Camkk2	calcium/calmodulin-dependent protein kinase kinase 2	4.0	−3.0	−3.7
Pgd	phosphogluconate dehydrogenase	5.2	−4.1	−5.3
Insig1	insulin induced gene 1	7.8	−7.2	−4.3

**Figure 2:**
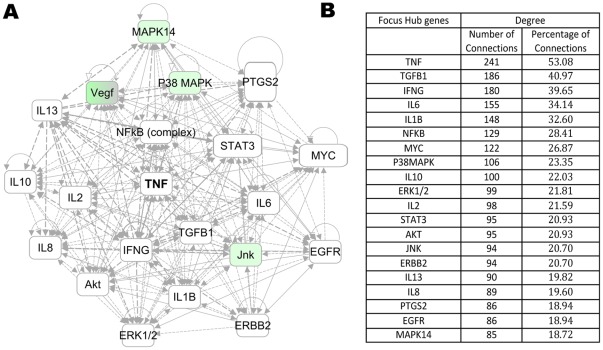
Counter-regulated network signature in PLNs. A) Interactive Network of top 20 focus gene hubs. B) Ranked list of top 20 focus gene hubs on the basis of degree of connectivity. A merged network was generated from the top 7 networks of AAT treatment counter-regulated genes in PLNs. The Ingenuity pathways analysis (IPA) tool was used to generate and merge the significantly effected networks from the AAT treatment counter-regulated genes. The focus gene hubs were ranked in the merged network on the basis of degree of connectivity.

**Figure 3:**
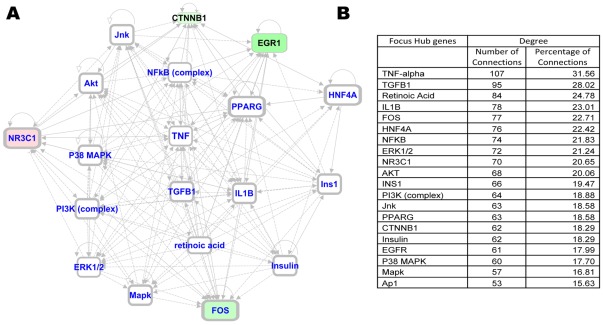
Counter-regulated network signature in fat. A) Interactive Network of top 20 focus gene hubs. B) Ranked list of top 20 focus gene hubs on the basis of degree of connectivity. The interactive network based analysis on the 238 fat counter-regulated genes identified 5 significant networks (Score≥40) related to cell cycle, lipid metabolism and carbohydrate metabolism. The merged network was generated from the top 3 networks of genes counter-regulated in fat by AAT and PM treatment. The focus gene hubs were ranked in the merged network on the basis of degree of connectivity.

To gain further insight into the impact of AAT treatment on PLNs, we performed systems biology oriented analysis on the 1,367 transcripts showing an AAT induced reversal pattern, i.e., genes whose expression in PLNs resembles those of normal mice following, but not before treatment, using Ingenuity Pathway Analysis (IPA) 5.0 (www.ingenuity.com). This approach enabled us to identify 10 interactive networks of genes with scores >15. The network score is an indicator of significance, the higher the score the more significantly the biological network is perturbed. To understand the underlying biological mechanisms, we merged the top 7 networks including genes related to inflammatory response and cellular growth into one network. This interactive network analysis incorporated additional genes into the network that were not identified as differentially expressed in the counter-regulation analysis (shown in white color). Several of these genes formed regulatory or highly connected nodes that serve as focus hubs in the networks, suggesting that these genes are targeted by many other genes. The merged network was analyzed to identify network hubs and bottlenecks, which may represent the key regulatory nodes in the network. The key hubs in the network were identified using degree of connectivity (number of interactions for a node with other network genes). An interactive network of the top 20 ranked focus gene hubs is shown in Figure 2A. The detailed network of focus hubs and interacting genes counter by AAT treatment is shown in Figure S1. The focus hubs are formed by inflammation-regulated genes (e.g., TNF-α, NF-κB, TGF-β, IL6, IL-1β, VEGF), kinases (e.g. AKT, PI-3 Kinase, P38 MAPK), and cell cycle/proliferation related genes (ERK1/2, MYC) (**Fig. 2B**). Among these focus hub genes TNF-α is the most highly connected gene, accounting for 53% of the connections (**Fig. 2B**). Similar interactive network based analysis on the fat counter-regulated genes (238) identified 5 significant networks (Score≥40) related to cell cycle, lipid metabolism and carbohydrate metabolism. The merging of the three significantly effected networks generated a complex network with regulatory hubs formed by inflammation related genes (e.g. TNF-α, NF-κB), kinases (e.g. AKT, PI-3 kinase), and transcriptional regulators (HNF4A, EGR1, Jnk) as well as diabetes related genes (Insulin, Ins1) (**Fig. 3**). The top 20 ranked focus gene hubs are shown in Figure 3A. The detailed network of focus hubs and interacting genes counter by AAT and PW in fat is shown in Figure S2. The focus gene hubs are likely critical for overall function of the network and, thus, interruption of such genes by therapeutic intervention is anticipated to perturb the whole network of genes. Interestingly, interactive network based analysis identified significant hubs formed by TNF-α, NF-κB, AKT, and PI-3 kinase in both fat and PLNs. TNF-α again emerged as the highest connected gene, accounting for 32% of the connections. In fat and PLNs the treatments apparently result in perturbation of similar and critical functional modules that are associated with curative effects. TNF-α was identified as the highest connected node in both PLNs and fat, respectively, suggesting its critical role in curative effects.

**Table 2:** Short-term treatment of Diabetic NOD mice with anti-TNF-α treatment permanently restores euglycemia.

Treatment	Normoglycemia achieved (range in days)	Normoglycemic (%)	Total number of mice used
NONE	N/A	0%	150 (Historical controls)
NONE	N/A	0%	20 (New controls)
Control mAb	N/A	0%	10
Anti-TNF-α	1–38	92%	24

### Short-term anti-TNF-α treatment restores an enduring euglycemic state in new onset diabetic NOD mice

As noted above, the potential role of TNF-α in the pathogenesis of diabetes is interesting and somewhat controversial. As our array and pathways oriented approach (see above) suggested TNF-α as the top focus gene hub, it is a potential therapeutic target linked to NF-κB directed inflammation, another significant hub. Thus, we tested the efficacy of a short course of anti-TNF-α in new onset (>10 days) T1D/T2D NOD mice whose thrice-repeated blood glucose levels ranged from 200 to 350 mg/dl. All untreated diabetic NOD mice remained hyperglycemic despite daily insulin therapy without spontaneous remissions (**Table 2**) and most died within 7 weeks of onset of T1D. In contrast, long lasting (>200 days follow up) euglycemia (80–160 mg/dl) was rapidly (1–38 days) achieved in 22 of 24 anti-TNF-α, but not control, mAb treated diabetic NOD mice despite cessation of anti-TNF-α therapy (**Table 2**).

**Table 3:** Short-term treatment of Diabetic NOD mice with anti-TNF-α therapy restores immune tolerance to beta cells.

Group	DONOR (islets)	RECIPIENTS	TREATMENT	SURVIVAL (days)	Number of mice used
A	NOD-scid	NOD-sp	NONE	4–21	5
B	C57BL/6	NOD-sp	NONE	5–8	20
C	NOD-scid	NOD-sp/stz*	240–300 days after anti-TNF-α treatment	>70	11
D	C57BL/6	NOD-sp/stz*	240–300 days after anti-TNF-α treatment	3–11	7

NOD-sp spontaneous new onset diabetic NOD mice;

* NOD-sp/stz, a streptozotocin induced diabetic state was induced in NOD recipients. Spontaneously diabetic NOD mice (**Groups C, D**) were previously restored to a euglycemic state by anti-TNF-α treatment. These mice remained euglycemic 240–300 days following the cessation of treatment.

Syngeneic NOD.SCID islet (**Groups A, C**) or allogeneic C57BL/6 (**Groups B, D**) islet grafts were transplanted into NOD recipients.

### Anti-TNF-α treatment induces immune tolerance selective to syngeneic beta cells in T1D NOD mice

As shown in **Table 3**, control untreated spontaneously new onset T1D NOD recipients reject Non-Obese Diabetic- Severe Combined Immunodeficiency mouse (NOD-scid) syngeneic or C57BL/6 allogeneic islet grafts and become diabetic 4–21 or 5–8 days, respectively, post-transplantation (**Table 3** **, Groups A and B**). To determine whether euglycemic anti-TNF-α treated spontaneously new onset T1D NOD mice were rendered tolerant to their islets, we destroyed remnant beta cells through administration of streptozotocin (STZ) long-following (240–300 days) cessation of anti-TNF-α therapy (**Table 3** **, Groups C and D**). Subsequently syngeneic or allogeneic islet grafts were transplanted into successfully treated NOD mice whose diabetic state was rekindled with STZ administration (**Table 3** **, Groups C and D**). Without re-institution of immunosuppressive therapy in hosts previously treated with anti-TNF-α, all STZ treated recipients of syngeneic (**Table 3** **, Group C**), but not allogeneic (**Table 3** **, Group D**), islets became normoglycemic within 24 hours and remained normoglycemic thereafter. Allogeneic islets transplanted into spontaneously diabetic NOD mice treated with STZ are rapidly rejected (**Table 3** **, Group C**). Hence, anti-TNF-α treatment creates a drug-free tolerant state to syngeneic insulin producing beta cells.

### Islet histology, beta cells mass and circulating insulin levels

**Figure 4:**
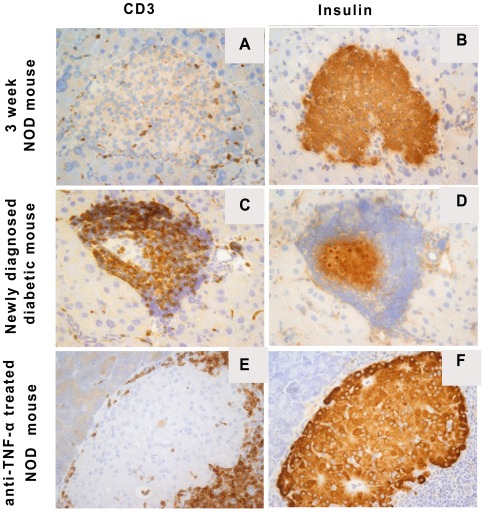
Immunohistochemical staining for CD3 and insulin in NOD pancreatic islets from different experimental groups. Representative frozen sections from a 3 week old female pre diabetic NOD mouse without insulitis (A, CD3; B, insulin), a newly diagnosed diabetic female mouse (C, CD3; D, insulin) and a diabetic NOD female mouse treated with anti-TNF-α (E, CD3; F, insulin). Overall the figures document increasing cellular infiltration by CD3+ T cells and the associated loss of insulin positive cells in diabetic (C, D), as compared to prediabetic, hosts (A, B). After anti-TNF-α treatment, intra-islet infiltration by CD3+ T cells is abolished with the appearance of a circumferential non-invasive CD3+ T cell process (E). After treatment many islets are found with normal insulin+ beta cells (F).

Previous morphometric analysis of the insulin positive mass of pancreatic islets revealed that NODs with blood glucose levels between 250 mg/dl–350 mg/dl have about 25% of the insulin positive beta cell mass of non-autoimmune NOD.SCID mice (5). The 25% residual beta cell mass is similar to that found in newly diagnosed patients with T1D. Histological and immunohistochemical analysis was performed on islets from young 3-week-old non-diabetic, age matched new onset diabetic NOD mice treated with insulin or insulin plus anti-TNF-α. Islets from young non-diabetic NOD mice revealed rare CD3+ mononuclear leukocytes within the islets and abundant insulin expressing beta cells (**Fig. 4** **A&B**). In new onset diabetic NOD mice a prominent intra- and peri-islet CD3+ T cell rich process and grossly diminished numbers of insulin staining beta cells are evident (**Fig. 4** **C&D**). Islets from anti-TNF-α treated new onset diabetic mice, similar to islets from non-diabetic NOD mice, show normal numbers of insulin expressing beta cells (**Fig. 4** **E&F**). Only peri-islet, not islet invasive, CD3+ T cells are present (**Fig. 4** **E&F**). Thus, successful treatment alters the insulitis from an invasive to a non-invasive circumferential pattern.

### Anti-TNF-α treatment ablates insulin resistance in new onset T1D/T2D NOD mice

**Figure 5:**
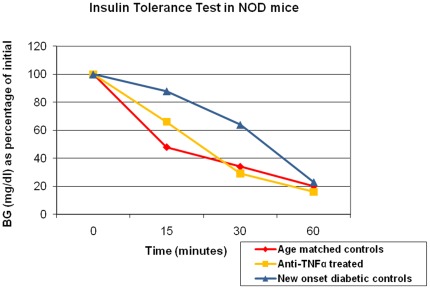
TNF-α treatment ablates insulin resistance in diabetic NOD mice. Insulin tolerance tests were performed in age matched NOD mice (n = 10/group): 1) new onset diabetic NOD mice; 2) anti-TNF-α treated new onset NOD mice; 3) age matched normoglycemic control NOD mice. Food was withheld 3 hours before testing. Animals were weighted and blood samples collected at 0 minutes, animals were injected i.p. with 0.75 U/kg of regular human insulin (Novolin, Novo Nordisk Pharmaceutical Industries, Inc. Clayton, NC). Blood samples were then collected at 15, 30 and 60 minutes. The blood glucose (BG) results were expressed as percentage of the baseline blood glucose concentration.

Recent studies have revealed insulin resistance in new onset diabetic NOD mice [3], [5]. Hence we sought to determine whether anti-TNF-α treatment restores the sensitivity of NOD mice to insulin driven disposal of blood glucose. Blood glucose levels in 10-week-old new onset diabetic mice fell only 37% over a 30 min period following an intraperitoneal injection of insulin, but decreased by ca. 80–85% in anti-TNF-α treated mice (**Fig. 5**). This pattern was also noted in age matched control non-diabetic NOD mice (**Fig. 5**). Thus, anti-TNF-α treatment ablates insulin resistance, thereby normalizing the response of host tissues to insulin.

### Anti-TNF-α treatment restores in vivo insulin signaling in diabetic NOD mice

**Figure 6:**
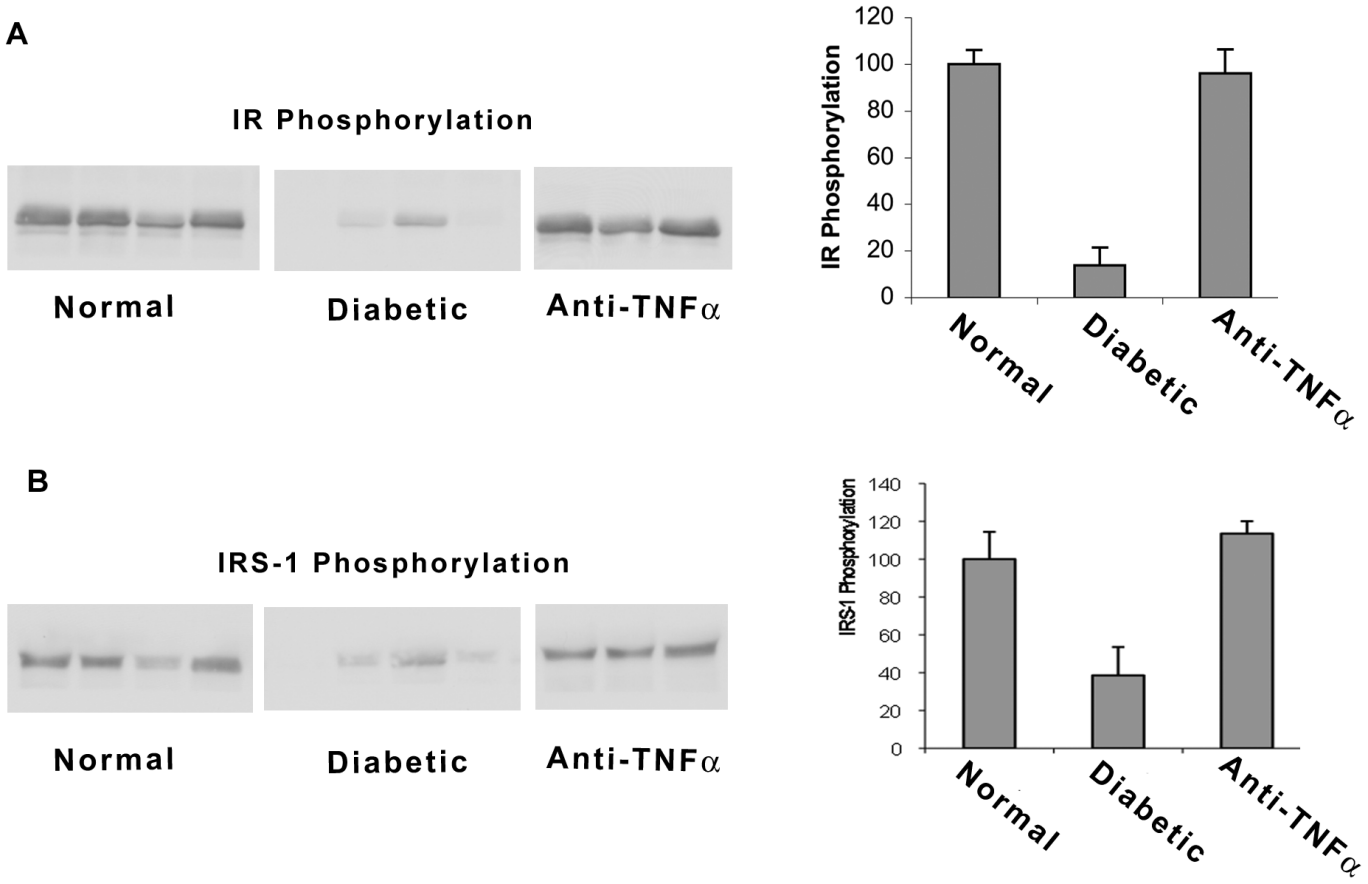
Anti-TNF-α treatment restores insulin signaling in new onset diabetic NOD mice. A) Compares tyrosyl phosphorylation of the insulin receptor in normal controls vs. newly diabetic and anti-TNF-α treated NOD mice. B) Compares tyrosyl phosphorylation of IRS-1 in normal controls vs. newly diabetic and anti-TNF-α treated NOD mice. The left side shows representative immunoblots. The right side shows quantitative analysis of the immunoblots. Mice (n = 6–8/group) were fasted overnight and injected with human insulin (20 units/kg body weight i.p.) to acutely stimulate insulin signaling. Mice were sacrificed 10 minutes later. Fat tissue obtained (50 days post-treatment) was dissected and frozen in liquid nitrogen before immunoblotting analysis of insulin signaling proteins among i) normal non-diabetic NOD mice; ii) newly diagnosed diabetic NOD mice treated with conventional insulin therapy; iii) anti-TNF-α treated NOD mice at 50 days post- treatment.

As insulin resistance in new onset diabetic NOD mice is accompanied by defective in vivo insulin signaling in tissues that are targeted for glucose disposal [4], [5], we examined the effects of anti-TNF-α upon insulin signaling in fat of new onset diabetic NOD mice. Insulin-stimulated tyrosyl phosphorylation of the insulin receptor (IR) was markedly (90%) diminished in new onset T1D NOD mice, as determined by immunoblot densitometry, compared to age matched control non-diabetic NOD mice (**Fig. 6A**). Impaired insulin signaling was also evident with respect to insulin-stimulated tyrosyl phosphorylation of insulin receptor substrate-1 (IRS-1) (**Fig. 6B**), a molecule that normally transmits the downstream signals of the insulin activated IR. The impact of short term anti-TNF-α therapy upon tyrosyl phosphorylation patterns in new onset T1D mice rendered euglycemic by anti-TNF-α therapy was compared with that obtained with mice rendered euglycemic from the time of diagnosis of overt diabetes with intense insulin therapy delivered i.p. with osmotic pumps. Anti-TNF-α therapy, unlike osmotic insulin pump therapy, does not immediately render the treated mice euglycemic. We have previously demonstrated that osmotic insulin pump therapy while correcting hyperglycemia does not restore normal insulin-triggered tyrosyl phosphorylation patterns [4]. Treatment with anti-TNF-α, but not intense osmotic pump delivered insulin or conventional insulin, completely restored the tyrosyl phosphorylation of IR and IRS-1 in new onset T1D NOD mice. Anti-TNF-α treatment apparently ablates insulin resistance via restoration of normal tyrosyl phosphorylation dependent insulin signaling.

## Discussion

Short-term treatment with PM [5] or with AAT [4] permanently restores euglycemia, self-tolerance to islets, and also restores effective insulin sensitivity/signaling [4], [5]. As a means to search for new therapeutic targets, we have applied genome wide transcriptional profiling, systems biology, and pathway analysis techniques to further examine the curative impact of PM [5] and AAT [4] regimens upon pancreatic lymph nodes, a relevant immune system site and fat, a site for insulin directed glucose disposal, in new onset T1D/T2D NOD mice. Systems biology analysis of the transcriptional profiles of animals treated with PM or AAT identified TNF-α as the top focus gene hub, as determined by the highest degree of connectivity, in both tissues. In PLNs and fat TNF-α interacted with 53% and 32% of genes, respectively, associated with reversal of diabetes by previous treatments. In short, our molecular analysis suggested that PM [5] and AAT [4] both may act in part by quenching a detrimental TNF-α dependent effect in both fat and PLNs. Some investigators advocate therapy with TNF-α or TNF-α inducers [15], [16] as treatments for autoimmune diseases including T1D. Nonetheless, we administered neutralizing anti-TNF-α mAb short term to new onset diabetic NOD mice. Gratifyingly, anti-TNF-α mAb administration to new onset T1D/T2D NOD mice served to enduringly restore euglycemia, self-tolerance, and normal insulin signaling. While many treatments prevent the occurrence of diabetes in the NOD model, few permanently restore euglycemia and self-tolerance. Indeed anti-TNF-α has been reported to prevent diabetes but not restore euglycemia in overtly diabetic NODs [12]. Our protocol for strict diabetes control, a protocol not used in the previous study [12], was crucial to success as an adjunct to anti-TNF-α treatment.

Previous studies have shown that an elevation of TNF-α levels during the neonatal period in NOD mice increases the frequency and hastened onset of T1D [13], [14]. Injection of neutralizing anti-TNF-α into newborn NOD mice results in complete prevention of disease [13]. TNF-α may function in part by activating macrophages. As a consequence, activated macrophages may enter the islets and begin to (i) recruit auto-reactive lymphocytes [17], [18]; (ii) process and present beta cell auto-antigens; and (iii) release pro-inflammatory cytokines that promote effector type responses by autoreactive T cells. That subtle inflammation, including critical expression of TNF-α, is associated with and likely causal for obesity linked T2D insulin resistance and faulty insulin signaling is well appreciated [19]. Our new observations indicate that a similar pattern of inflammation exists in both insulin sensitive (fat) and pancreatic lymph nodes of new onset diabetic NOD mice in which TNF-α is an important hub related to the pathogenesis of T1D and T2D. Our data do not prove that TNΦ–α per se causes diabetes, but the data, in keeping with the bioinformatics analysis showing this molecule as a major hub in the disease, prove that in the NOD mouse, diabetes is a dependent process. One can speculate the rapid restoration of euglycemia was enabled by the ablation of insulin resistance and ablation of the pro-inflammatory invasive insulitis enabling dysfunction but not destroyed beta cells to resume insulin production. It is notable that a small pilot trial with etanercept, a soluble TNF-α receptor Ig fusion protein that binds to TNF-α, shows promise as a treatment for children with new onset T1D [20]. Overall this study re-enforces the view that pro-inflammatory cytokines play a cardinal role in T1D [4], [5].

This study emphasizes the potential importance of analyzing molecular pathways as a means to identify potential therapeutic targets. There is a vast literature concerning the beneficial therapeutic effects achieved by neutralizing TNF-α with antibodies or circulating receptor proteins in a variety of autoimmune states [21]. Neutralization of TNF-α in these states leads to profound effects upon TNF-α sensitive pro-inflammatory cytokine cascades [21]. In this sense, the beneficial consequences of anti-TNF-α in the clinically relevant new onset T1D NOD model might have been foreseen despite suggestions that (i) TNΦ–α inducers might prove therapeutic and (ii) TNF-α neutralizing therapy might prove detrimental [15], [16].

## Methods

### Ethics Statement

All mice were maintained under pathogen-free conditions at the Harvard Institutes of Medicine (Boston, MA). The Harvard Medical School institutional review board approved all animal studies. The approved protocol number is #03827.

### Mice

Female NOD (NOD/LtJx) mice and NOD.SCID (NOD.CB17-Prkdc^scid^/J) mice were purchased from Jackson Laboratories (Bar Harbor, ME) at 4 wks of age.

### Analysis of Gene Expression Data

Transcriptional profiles of fat and PLNs in normoglycemic NOD mice (NOR), new onset diabetic (DIA), new onset diabetic NOD mice treated with AAT or PM were characterized using the Mouse 430 2.0 Affymetrix GeneChip, according to previously described protocols for total RNA extraction and purification, cDNA synthesis, in vitro transcription reaction for production of biotin-labeled cRNA, hybridization of cRNA with mouse 430 2.0 Affymetrix gene chips, and scanning of image output files [22]. Raw data was submitted to NCBI Gene Expression Omnibus (GEO) database (GEO accession number GSE33891). All experiments were performed at least in duplicate on the fat and PLN NOD tissue samples. The chip quality was determined using the SimpleAffy package of Bioconductor [23]. To obtain the signal values, chips were further analyzed using Robust Multichip Average (RMA) method in R using Bioconductor and associated packages. RMA performed the background adjustment, the quantile normalization and final summarization of 11 oligonucleotides per transcript using the median polish algorithm. When comparing normal vs. diabetic mice, we used a non-parametric method (RankProd) implemented as a Bioconductor package for identification of differentially expressed genes [24]. For identification of differentially expressed transcripts, P values were obtained based on 100 random permutations in the RankProd package. The genes that had P values less than or equal to 0.05 and absolute fold change (FC) >2 between the normal and diabetic mice were considered as differentially expressed.

To identify genes that are involved in the reversal of diabetes after treatment with AAT or PM, we performed counter-regulation analysis on the transcripts that are differentially expressed between diabetic NOD and non-diabetic NOD (i.e., NOR) mice in fat and PLNs [4]. Counter-regulation means that treatment down-regulates the genes that are up-regulated in diabetic vs. normal mice and vice versa. K-means clustering of differentially expressed transcripts to 20 bins was performed to identify transcripts that are counter-regulated by treatments. Bins of transcripts manifesting different degrees of counter-regulation were identified and further filtered on the basis of magnitude and P value based significance of counter-regulation. The final list of counter-regulated genes was generated by considering genes that are counter-regulated by a magnitude of fold change (FC) >2 and P value<0.05 on treatment as compared to diabetic mice.

Interactive network, pathway and function analyses were performed on significantly counter-regulated genes using Ingenuity Pathways Analysis (IPA 5.0) (www.ingenuity.com), a systems biology oriented package. The knowledge base of this software consists of functions, pathways and network models derived by systematically exploring the peer reviewed scientific literature. This database consisting of millions of individually modeled relationships between proteins, genes, cells, tissues, drugs, and diseases for the identification of key functions and pathways distinguishing biologic states. A detailed description of IPA analysis is available at the Ingenuity Systems' web site (http//www.ingenuity.com). IPA calculates the P value using Fisher's Exact Test for each pathway and function according to the fit of user's data to the IPA database. The P value measures how likely the observed association between a specific pathway/function/interactive network and the data set would be if it was only due to random chance, by also considering the total number of Function/Pathway/Interactive Network eligible genes in the test dataset and the reference sets of genes. The focus molecules were identified from the integrated networks on the basis of degree of connectivity (number of interactions for each gene). The focus hubs with higher degrees of connectivity are considered critical for maintenance of the networks, suggesting that therapeutic targeting of these focus hubs may elicit the strongest impact.

### Blood glucose

Blood glucose levels of NOD mice were monitored 2x/wk with the Accu-Check blood glucose monitor system (Roche, Indianapolis, IN). When non-fasting blood glucose levels are in excess of 200 mg/dl on three consecutive measurements a diagnosis of new onset of diabetes is made. In practice, only mice with blood glucose levels between 250 to 350 mg/dl became subjects in the anti-TNF-α trial. Why? Previous morphometric analysis of the insulin positive mass of the pancreatic islets revealed that NODs with blood glucose levels between 250 mg/dl–350 mg/dl have about 25% of the insulin positive beta cell mass of non-autoimmune NOD.SCID mice [5]. The 25% residual beta cell mass is similar to that found in newly diagnosed patients with T1D [25].

### Treatment protocols

Individualized insulin therapy was employed and doses were calibrated on the basis of three blood glucose measurements per day. The goal was to maintain blood glucose levels between 100–160 mg/dl. For blood glucose levels in excess of 160 mg/dl, NPH insulin is given in doses ranging 1 to 4 International Units depending upon the magnitude of hyperglycemia. Mice with high blood glucose levels in excess of 400 mg/dl for 2 days also received 0.2–0.4 ml warm normal saline subcutaneously. For syngeneic islet transplant recipients, blood glucose levels were checked at the time of transplantation, then daily for 2 wks, and then 2–3x/wk.

The hamster clone IgG1 (TN3-19.12; Sigma St Louis, MO) anti-TNF-α monoclonal antibody (mAb) was given to new onset diabetic NOD mice at a dose of 100 mg intraperitoneal every other day for 10 doses. Administration of an irrelevant hamster isotype (hamster IgG1 clone G235-2356; BD, San Jose, CA) for the same duration and dose served as a control treatment.

### Streptozotocin (STZ) induction of diabetes

β-cells were destroyed in formerly spontaneously diabetic NOD mice in which anti-TNF-α treatment rendered mice euglycemic by administration of streptozotocin (STZ; 275 mg/kg intraperitoneal), a beta cell toxin. STZ was administered between 220 to 320 days following the restoration of euglycemia and long following cessation of anti-TNF-α treatment. With the re-emergence of hyperglycemia following STZ administration, these diabetic NOD mice were transplanted with syngeneic or allogeneic islets in the absence of immunosuppressive therapy. Graft failure was defined as the first day of 3 consecutive days of blood glucose levels >250 mg/dl.

### Islet transplantation

NOD.SCID or C57BL/6 mice (10–12 wks old) were used as donors for islet transplants. After islet purification, islets with diameters between 75 and 250 µm were hand picked and transplanted under the renal capsule [26]. Each recipient received 600–800 NOD.SCID or C57BL/6 islets.

### Insulin tolerance test

Insulin tolerance tests were performed as previously described [4], [5] in age matched NODs including 1) spontaneous new onset diabetic NOD mice (NOD-sp); 2) anti-TNF-α treated spontaneous new onset diabetic NOD mice (NOD-sp); and 3) non-diabetic NOD mice. Blood samples were collected at 15, 30 and 60 minutes after insulin injections with results expressed as a percentage of the initial blood glucose concentration [4], [5].

### In vivo insulin signaling studies

In vivo insulin signaling experiments were performed as previously reported [4], [5] on mice after a 16 hr fast. Mice were injected i.p. with 20 U/kg of human regular insulin (Eli Lilly, Indianapolis, IN) or saline and sacrificed 10 minutes later. Skeletal muscle (gastrocnemius), fat (epididymal white adipose tissue) and liver were dissected and frozen in liquid nitrogen for immunoblotting analysis of insulin signaling proteins and purification of RNA. Immunoblotting for insulin receptor and IRS-1 phosphorylation was performed as previously described [4], [5].

### Analysis of beta cell mass

Islet sections (5 µm) were immunostained (peroxidase-antiperoxidase) using rabbit anti-bovine glucagon (1∶3000, gift of Dr. M. Appel) or anti-insulin (1∶200, Linco, Billerica, MA). Beta cell mass was measured by point counting morphometry and beta cell relative volume (intercepts over beta cells divided by intercepts over total pancreatic tissue) was multiplied by the pancreas weight to calculate the beta cell mass [27].

### Analysis of pancreas infiltrating CD3+ T cells

Pancreases excised from anesthetized mice (non-diabetic, newly diabetic and anti-TNF-α treated) were analyzed were fixed in Bouin's solution and embedded in paraffin or frozen sections. Five-micron paraffin sections were mounted on charged Superfrost Plus microscopy slides (Erie Scientific Company Portsmouth, NH), air-dried overnight, and incubated at 56°C for 15 minutes. Sections were then deparaffinized, and incubating slides in PBS solution containing 0.3% hydrogen peroxide blocked endogenous peroxidase activity. After blockade of “non-specific” binding with horse serum, insulin staining with insulin primary antibody (cat#A0564 Dako Corporation, Carpinteria, Calif., USA) was performed on the sections. To identify CD3+T cells, the sections were treated with heat mediated antigen retrieval (10 mM sodium citrate) for 1 hr, followed by “non-specific” blocking with horse serum. This was followed by overnight incubation at 4°C with the primary antibody (CD3 antibody; cat#MCA1477 Serotec). Staining procedures continue with application of biotin labeled secondary antibodies (mouse anti-guinea pig cat# BA7000, mouse anti-rat cat# BA4001 Vector Lab Inc., Burlingame, CA), and the signal was enhanced with avidin-biotin complex-HRP (Elite Standard, cat# PK-6100 from Vector Lab. Inc., Burlingame, CA). The signal was visualized with ImmPACT DAB kit (cat# SK-4105 Vector Lab. Inc., Burlingame, CA) and counter stained with Gill's hematoxylin II. The sections were analyzed with Olympus BX51 microscope, DP71 camera sot program.

## Supporting Information

Figure S1 **Interactive Network representation of counter regulated genes in PLNs.** The merged network was generated from top 7 networks of AAT treatment counter regulated genes in PLNs. The ingenuity pathways analysis (IPA) tool was used to generate the networks from the AAT treatment counter regulated genes and for merging the significantly effected networks. The intensity of the node color indicates the degree of up-regulation (red) and down-regulation (green) in treated mice as compared with the diabetic PLNs. Top 20 Focus hubs are highlighted in the network.
(JPG)

Figure S2 **Interactive Network representation of AAT and PM treatment counter regulated counter regulated genes in fat.** The interactive network based analysis on the fat counter regulated genes (238) identified 3 significant networks (Score≥40) related to cell cycle, lipid metabolism and carbohydrate metabolism. The ingenuity pathways analysis (IPA) tool was used to generate the networks from the AAT and PW treatments counter regulated genes and for merging the significantly effected networks. The intensity of the node color indicates the degree of up-regulation (red) and down-regulation (green) in treated mice as compared with the diabetic PLNs. Top 20 Focus hubs are highlighted in the network.
(JPG)
