# Supplementary figures and images for: The Role of TNF-α in Mice with Type 1- and 2- Diabetes
Source: PLoS One. 2012 May 11;7(5):e33254. doi: 10.1371/journal.pone.0033254 (PMC3350520; doi:10.1371/journal.pone.0033254)

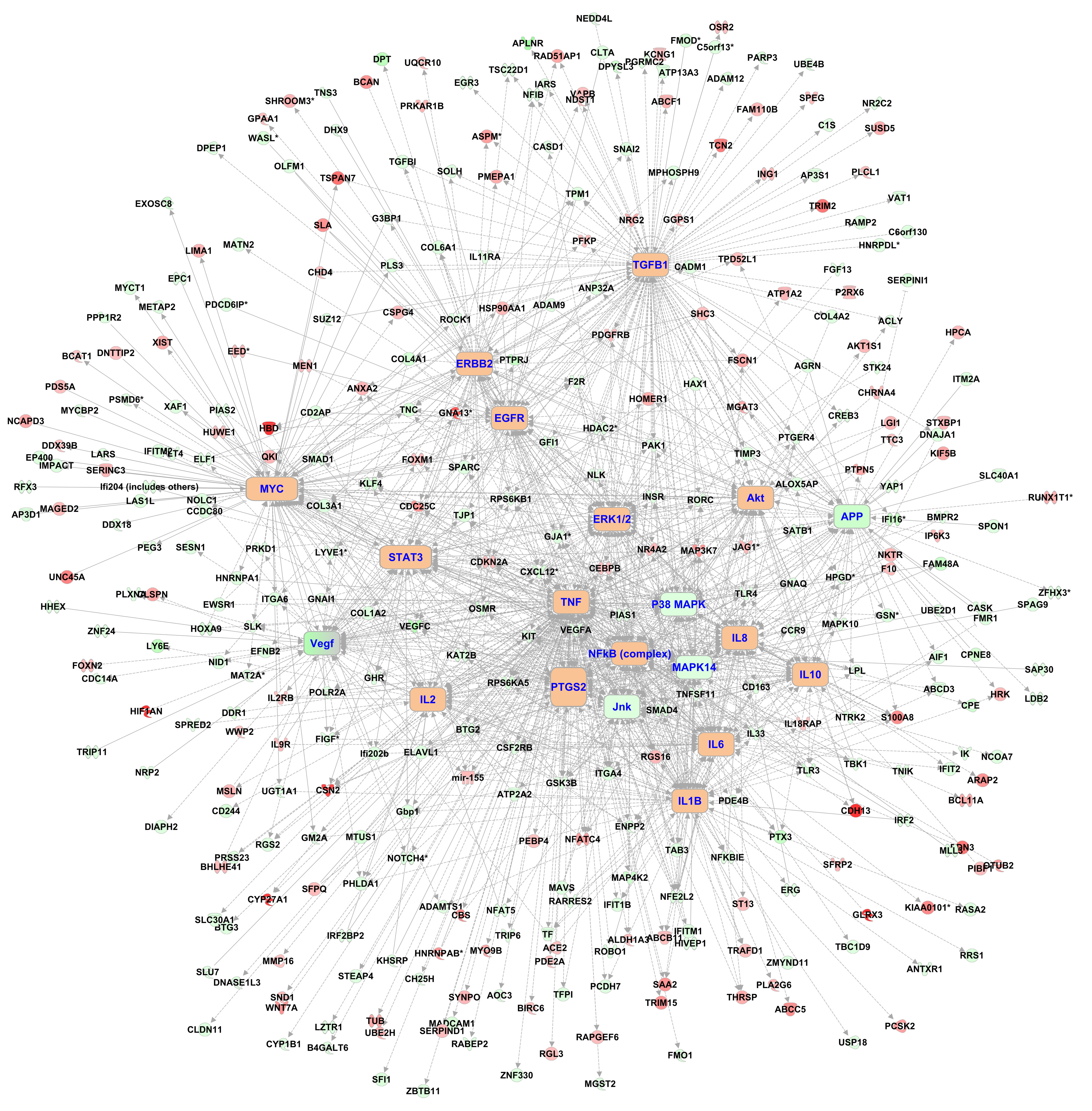

Supplement: Figure S1 — Interactive Network representation of counter regulated genes in PLNs. The merged network was generated from top 7 networks of AAT treatment counter regulated genes in PLNs. The ingenuity pathways analysis (IPA) tool was used to generate the networks from the AAT treatment counter regulated genes and for merging the significantly effected networks. The intensity of the node color indicates the degree of up-regulation (red) and down-regulation (green) in treated mice as compared with the diabetic PLNs. Top 20 Focus hubs are highlighted in the network. (JPG) [file pone.0033254.s001.jpg]

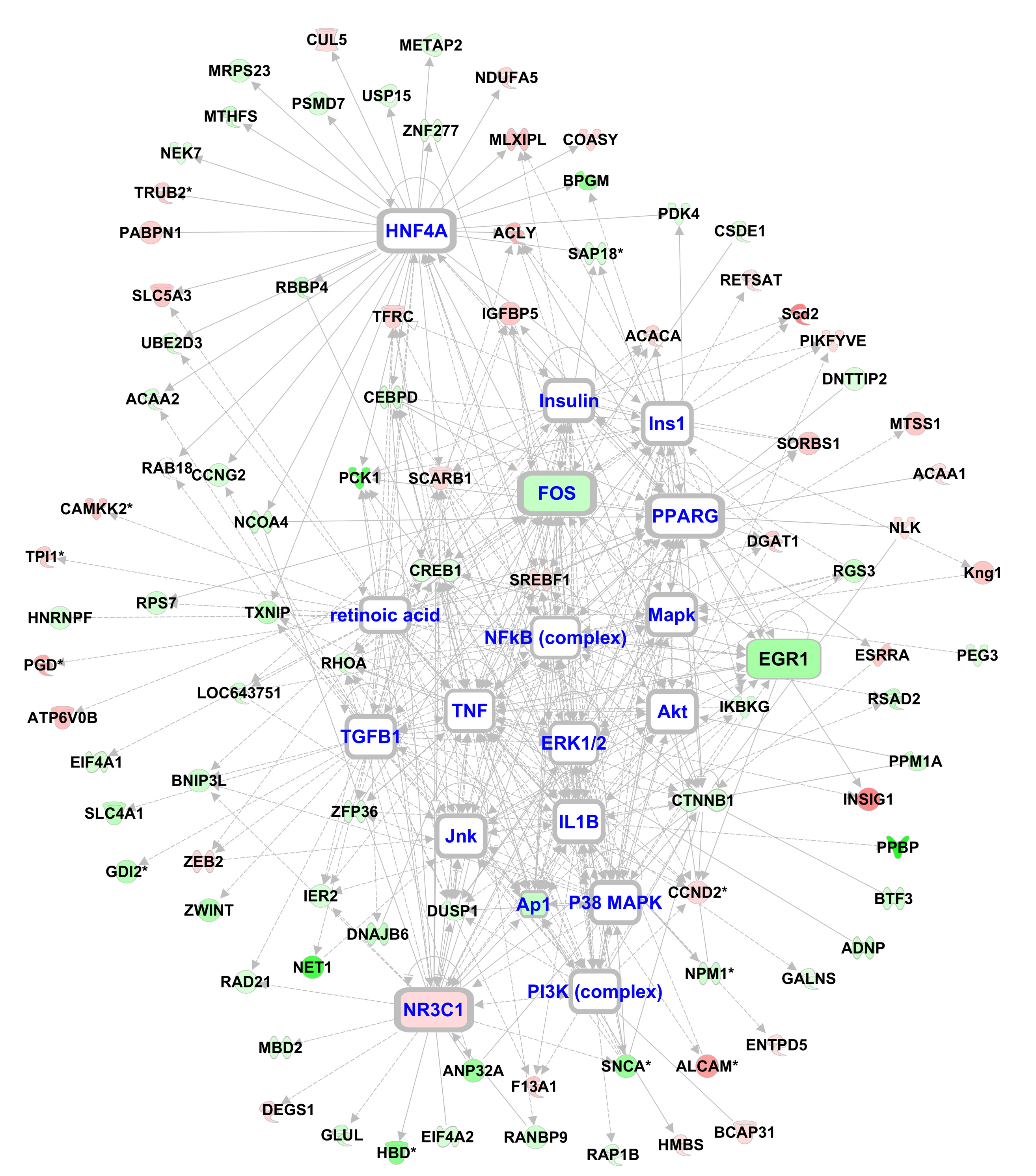

Supplement: Figure S2 — Interactive Network representation of AAT and PM treatment counter regulated counter regulated genes in fat. The interactive network based analysis on the fat counter regulated genes (238) identified 3 significant networks (Score≥40) related to cell cycle, lipid metabolism and carbohydrate metabolism. The ingenuity pathways analysis (IPA) tool was used to generate the networks from the AAT and PW treatments counter regulated genes and for merging the significantly effected networks. The intensity of the node color indicates the degree of up-regulation (red) and down-regulation (green) in treated mice as compared with the diabetic PLNs. Top 20 Focus hubs are highlighted in the network. (JPG) [file pone.0033254.s002.jpg]
